# Use of standardised patients to assess antibiotic dispensing for tuberculosis by pharmacies in urban India: a cross-sectional study

**DOI:** 10.1016/S1473-3099(16)30215-8

**Published:** 2016-11

**Authors:** Srinath Satyanarayana, Ada Kwan, Benjamin Daniels, Ramnath Subbaraman, Andrew McDowell, Sofi Bergkvist, Ranendra K Das, Veena Das, Jishnu Das, Madhukar Pai

**Affiliations:** a McGill International Tuberculosis Centre & Department of Epidemiology, Biostatistics and Occupational Health, McGill University, Montreal, QC, Canada; b Manipal McGill Center for Infectious Diseases, Manipal University, Manipal, India; c Development Research Group, The World Bank, Washington, DC, USA; d Division of Infectious Diseases, Brigham and Women's Hospital and Harvard Medical School, Boston, MA, USA; e ACCESS Health International, Hyderabad, India; f Institute for Social and Economic Research on Development and Democracy, Delhi, India; g Department of Anthropology, Johns Hopkins University, Baltimore, MD, USA; h Center for Policy Research, New Delhi, India

## Abstract

**Background:**

India's total antibiotic use is the highest of any country. Patients often receive prescription-only drugs directly from pharmacies. Here we aimed to assess the medical advice and drug dispensing practices of pharmacies for standardised patients with presumed and confirmed tuberculosis in India.

**Methods:**

In this cross-sectional study in the three Indian cities Delhi, Mumbai, and Patna, we developed two standardised patient cases: first, a patient presenting with 2–3 weeks of pulmonary tuberculosis symptoms (Case 1); and second, a patient with microbiologically confirmed pulmonary tuberculosis (Case 2). Standardised patients were scheduled to present each case once to sampled pharmacies. We defined ideal management for both cases a priori as referral to a health-care provider without dispensing antibiotics or steroids or both.

**Findings:**

Between April 1, 2014, and Nov 29, 2015, we sampled 622 pharmacies in Delhi, Mumbai, and Patna. Standardised patients completed 1200 (96%) of 1244 interactions. We recorded ideal management (defined as referrals without the use of antibiotics or steroids) in 80 (13%) of 599 Case 1 interactions (95% CI 11–16) and 372 (62%) of 601 Case 2 interactions (95% CI 58–66). Antibiotic use was significantly lower in Case 2 interactions (98 [16%] of 601, 95% CI 13–19) than in Case 1 (221 [37%] of 599, 95% CI 33–41). First-line anti-tuberculosis drugs were not dispensed in any city. The differences in antibiotic or steroid use and number of medicines dispensed between Case 1 and Case 2 were almost entirely attributable to the difference in referral behaviour.

**Interpretation:**

Only some urban Indian pharmacies correctly managed patients with presumed tuberculosis, but most correctly managed a case of confirmed tuberculosis. No pharmacy dispensed anti-tuberculosis drugs for either case. Absence of a confirmed diagnosis is a key driver of antibiotic misuse and could inform antimicrobial stewardship interventions.

**Funding:**

Grand Challenges Canada, Bill & Melinda Gates Foundation, Knowledge for Change Program, and World Bank Development Research Group.

## Introduction

Antimicrobial resistance is a global health emergency,^1^, ^2^ and the indiscriminate use of antibiotics is a major driver.^3^, ^4^ Although India ranks first in total antibiotic use worldwide,^2^ the absence of data linking antibiotic use to underlying illnesses makes it hard to assess the appropriateness of such use in view of India's high infectious disease burden. With some of the highest incidences of drug-resistant bacterial pathogens in the world, identification of the sources and circumstances of antibiotic abuse as opposed to use is a crucial first step to understanding what can be done about antibiotic overuse in India.^4^ Here, we develop a unique method to address this gap, focusing our attention on a specific illness, tuberculosis, and a specific source of health care—pharmacies.

Our choice of tuberculosis, a disease that affects 2·2 million Indians every year, as a lens through which to investigate antibiotic use is driven by several factors. The symptoms of early pulmonary tuberculosis are common, non-specific, non-severe, and persistent. In this case, assessment of pharmacist behaviour provides a realistic and externally valid estimate of unnecessary antibiotic use. Further, indiscriminate drug use can harm both the patient and the efficacy of existing anti-tuberculosis treatments. For instance, tuberculosis symptoms subside temporarily with the use of fluoroquinolones or corticosteroids, delaying diagnosis and leading to the possibility that patients receive several antibiotic courses for the wrong diagnosis.^5^ Partial courses of anti-tuberculosis drugs can result in drug resistance.^6^ Finally, international and national guidelines for the optimum management of tuberculosis cases^7^, ^8^ allow the assessment of the extent of antibiotic misuse.

Our focus on pharmacies is premised on the belief that their practices contribute to the availability and use of antibiotics in the population.^4^ This premise is partly the result of their widespread availability—more than 750 000 private retail pharmacies provide easy access to drugs.^9^ However, our premise also reflects the willingness of pharmacists to provide prescription-only drugs to patients. Despite clear guidelines on the use of over-the-counter versus prescription-only drugs,^10^ enforcement is widely believed to be suboptimum.^11^, ^12^ Pharmacies are thought to be dispensing antibiotics and anti-tuberculosis drugs without prescriptions. Many tuberculosis patients do seek medical advice and drugs from pharmacies,^13^ driven by the ease of access and the possibility of avoiding consultation charges by doctors.^14^

**Research in context**

**Evidence before this study**

Antimicrobial resistance is a global health emergency, and, as the largest consumer of antibiotics, India is at highest risk. The standardised patient method can help to assess the extent and appropriateness of antibiotic use because such use can be directly related to the underlying illness of the patient. To identify previous research on this topic, we searched PubMed and Google Scholar using a combination of the terms “standardized patients” (“mystery clients”, “fake patients”, or “simulated patients”), “pharmacy” (“pharmacist” or “chemist”), and “tuberculosis” with and without the keyword “India” for articles published in English until March 31, 2015. Our search showed that previous studies of physician management of standardised patients in India have reported unnecessary antibiotic prescribing for various conditions, including tuberculosis, diarrhoea, asthma, and angina. However, these studies have not addressed antibiotic abuse by pharmacists, who respond to the health-care needs of a substantial proportion of India's population.

**Added value of this study**

We used standardised patients to quantify the extent of antibiotic overuse in pharmacists for patients with tuberculosis. We developed two standardised patient cases: first, a patient presenting with 2–3 weeks of pulmonary tuberculosis symptoms (Case 1); and second, a patient with microbiologically confirmed pulmonary tuberculosis (Case 2). Across all interactions, 319 (27%) of 1200 (95% CI 24–29) resulted in the use of an antibiotic although no pharmacy dispensed first-line anti-tuberculosis drugs. Ideal case management, defined as referrals without the use of antibiotics or steroids, was much lower in Case 1 interactions (13%) than Case 2 interactions (62%). Our study results add to the growing evidence on antibiotic abuse, but also underscore that the use and misuse of antibiotics are mediated by drug category and the information that patients present. Although antibiotic use is high and such use can delay diagnosis, none of the pharmacies dispensed first-line anti-tuberculosis drugs, and the use of stronger fluoroquinolone antibiotics and heavily restricted drug classes was low. Furthermore, the use of all antibiotics decreased sharply when the patient's diagnosis was made available to the pharmacists.

**Implications of all the available evidence**

Our findings suggest that non-adherence to regulatory standards is higher when the patient's condition is unknown, and that pharmacies prefer to treat in such cases rather than refer the patient to appropriate care. These findings can inform interventions to engage pharmacies in tuberculosis control and antimicrobial stewardship.

Tuberculosis is a major problem in all three cities studied (Delhi, Mumbai, and Patna), with notification rates (officially reported) of 294, 210, and 77 per 100 000, respectively.^15^ However, these rates are probably underestimated because many cases treated in the private sector are not notified.^16^ All three cities are experiencing rising rates of drug-resistant tuberculosis, especially in the city of Mumbai,^17^ and it is widely believed that pharmacists are a key component of the dispensing landscape and often a first contact for primary care.

Guidelines for pharmacies are specified under the Ministry of Health and Family Welfare's Drugs and Cosmetics Rules Act, 1945.^10^ All antibiotics and steroids are listed under two different schedules—Schedule H and Schedule H1. Schedule H drugs cannot be given to patients without a prescription from a qualified medical practitioner. In 2013, regulations were further tightened, with anti-tuberculosis drugs (isoniazid, rifampicin, ethambutol, and pyrazinamide) and some fluoroquinolones (such as moxifloxacin and levofloxacin, used in the treatment of tuberculosis) listed on a newly created Schedule H1. For H1 drugs, pharmacies require both a prescription from a qualified medical practitioner and a separate register to record the name and address of the prescriber, the patient, the names of the drugs and the quantity supplied.^18^ Schedule X, the most restrictive list, includes drugs such as narcotics, which require a prescription from a qualified provider to be retained by the retailer for 2 years.^19^

We have previously assessed the quality of tuberculosis care in India by health-care providers using standardised patients and use a similar method to study the practices of staff at pharmacies.^20^ Although standardised patients are routinely used to assess pharmacy practices in low-income and high-income countries,^21^ to our knowledge, no study has used standardised patients to assess pharmacy practices for tuberculosis in India. In our previous study, we validated the use of standardised patients for tuberculosis and showed the viability and accuracy of this method for measuring quality of tuberculosis care along several dimensions, including very low likelihood of detection, minimum to no study participation risk for either standardised patients or health-care providers, and high levels of accurate recall of the clinical interaction among standardised patients. This study complements our previous validation study by extending the method to pharmacists. The method developed here addresses the dual objectives of, first, assessment of pharmacists' behaviour and drug use for a patient with a complaint, but no prescription. Second, it allows us to assess how case management and drug use differs when the diagnosis is unknown versus confirmed.

## Methods

### Study design and setting

This cross-sectional study was done in Delhi, Mumbai, and Patna.

Through this multi-site study we aimed to assess the medical advice and drug dispensing practices of pharmacies for standardised patients presenting with either presumptive tuberculosis (Case 1) or microbiologically confirmed tuberculosis (Case 2). By assessing the difference in antibiotic use across the two cases for the same pharmacists, we broke down the relative importance of antibiotic misuse arising from the lack of diagnosis (Case 1) versus antibiotic use despite a confirmed diagnosis for which antibiotics are contraindicated (Case 2). To set the benchmark for what pharmacists should do when faced with such patients, we used guidelines from the Government of India and the Indian Pharmaceutical Association. These guidelines specify that pharmacies should counsel patients about tuberculosis, identify and refer persons with tuberculosis symptoms to the nearest public health facilities for tuberculosis testing, and play a part in the provision of tuberculosis treatment.^22^ Therefore, pharmacists adhering to these guidelines should have referred the standardised patients to health-care providers without dispensing either antibiotics or steroids, both of which require a prescription.

### Standardised patients

The two cases of standardised patients used in our study were adapted from our validation study in Delhi.^15^ Standardised patients trained as Case 1 presented with 2–3 weeks of cough and fever and were directly seeking drugs from a pharmacy. Differential diagnosis for this case included upper respiratory tract infection, pneumonia, asthma and acute or chronic bronchitis; antibiotic use might be warranted for some of these conditions although not without a prescription from a doctor.

Standardised patients trained as Case 2 presented with 1 month of cough and fever and a tuberculosis-positive laboratory report from a recent sputum smear test at a government dispensary. In this case, tuberculosis was confirmed, although the standardised patients, who presented as uninformed patients, made it clear that they did not fully understand what the report said. In this situation, the pharmacist plausibly could know the correct diagnosis and could recognise that short-term antibiotics would not help, but also could realise that the patient would still purchase antibiotics if offered because of their ignorance of the test results. Standardised patients did not present with drug prescriptions; table 1 shows their opening statements and case scenarios. After each pharmacy visit, standardised patients were debriefed with a structured questionnaire within 1 h of the visit. The accompanying appendix (pp 5,6) provides more details on the development of the cases and the recruitment and characteristics of the standardised patients in the study. Cases are available from the authors by request.

### Selection of pharmacies, standardised patient visits, and study size

Standardised patients visited 54 pharmacies in Delhi using a convenience sample from 28 low-income localities in April, 2014. This phase of the study validated the approach and provided key parameter estimates for power calculations employed in Mumbai and Patna. Based on these power calculations we sent standardised patients to 308 randomly sampled pharmacies in Mumbai and 260 in Patna between Nov 5, 2014, and Nov 29, 2015. 1200 (96%)of 1244 interactions were completed as planned, and we completed both cases for a sampled pharmacy in 1156 (93%) of 1244 scheduled interactions. The appendix discusses the sample and sampling weights, case development, standardised patient recruitment, sample size calculations, drug identification, and deviations from the sampling scheme (pp 2–8).

We obtained approvals from the ethics committees of McGill University Health Centre in Montreal, Canada, and the Institute of Socio-Economic Research on Development and Democracy (ISERDD) in New Delhi. Both ethics committees approved a waiver from obtaining informed consent from pharmacies in Mumbai and Patna. All individuals who participated as standardised patients were hired as staff and trained to protect themselves from any harmful medical interventions, such as avoiding injections, invasive tests, or consuming any drugs at the pharmacy.

### Statistical analysis

Our unit of analysis was a pharmacy-standardised patient interaction irrespective of who (pharmacy owners, pharmacists, or pharmacy assistants) the standardised patient interacted with. Whether the case was correctly managed was assessed from a tuberculosis perspective, consistent with Standards for Tuberculosis Care in India and International Standards for Tuberculosis Care.^7^, ^8^ We regarded ideal management for both cases as verbal or written referral to a health-care provider (public or private), without dispensing any antibiotics, including anti-tuberculosis drugs and fluoroquinolones, or steroids (table 1).

We calculated the proportion and 95% CI for our primary outcome, the proportion of interactions that resulted in ideal management, as well as the proportion of interactions resulting in antibiotic, fluoroquinolone, and steroid use with appropriate sampling weights (appendix p 3).

To assess the difference in case management and the use of drugs across the two cases, we used a random intercept logit model with indicator variables for each city as additional controls. In view of the study design and since every sampled pharmacy was attempted by both cases, the choice of model (logit, logit with fixed effects, or logit with random intercepts) should have yielded similar unbiased estimates, with differences arising only from the small portion of pharmacies that received one case but not the other. However, coefficients from the random-intercepts model are more precisely estimated. The appendix (pp 10–13) provides a series of alternate estimates, with both marginal effects and odds ratios from different model specifications and confirm that the results are very similar across specifications. All analyses were done using Stata (version 13).

### Role of the funding source

The funders of the study had no role in study design, data collection, data analysis, data interpretation, or writing of the report. The corresponding author had full access to all the data in the study and had final responsibility for the decision to submit for publication.

## Results

96 (16%) of 599 pharmacies (95% CI 13–19) referred Case 1 interactions to health-care providers, but because in 16 (17%) of these 96 cases the standardised patient was also given an antibiotic or steroid (95% CI 11–25), ideal case management (referral to a health-care provider without any antibiotics and steroids) occurred in 80 (13%) of 599 Case 1 interactions (95% CI 11–16). Overall, antibiotics were used in 221 (37%; 95% CI 33–41) of 599 interactions, steroids in 45 (8%; 95% CI 6–10), and fluoroquinolones in 61 (10%; 95% CI 8–13). Because Schedule H drugs also include prescription-only drugs that are not antibiotics or steroids (eg, ibuprofen or cetirizine), the use of these drugs was higher (401 [67%] of 599 interactions, 95% CI 63–71). The use of Schedule H1 drugs was notably lower (37 [6%] of 599, 95% CI 4–8) and Schedule X drugs and anti-tuberculosis drugs were never given. Table 2 provides the mean proportions of the key outcome variables in all cities combined for Case 1 and Case 2. Since the sampling scheme was different for Delhi compared with Mumbai and Patna, we also provide results excluding Delhi (table 2), and for each city by case (appendix pp 8,9).

By contrast with Case 1, 401 (67%) of 601 pharmacies (95% CI 63–70) referred Case 2 to a health-care provider (table 2). As before, some patients received antibiotics or steroids even with a referral, so ideal case management was recorded in 372 (62%) of 601 interactions (95% CI 58–66). Antibiotics, steroids, and fluoroquinolones were all used much less frequently, although Schedule H drugs were still given in 188 (31%) of 601 interactions (95% CI 28–35). As before, Schedule X and anti-tuberculosis drugs were never used.

**Figure 1:**
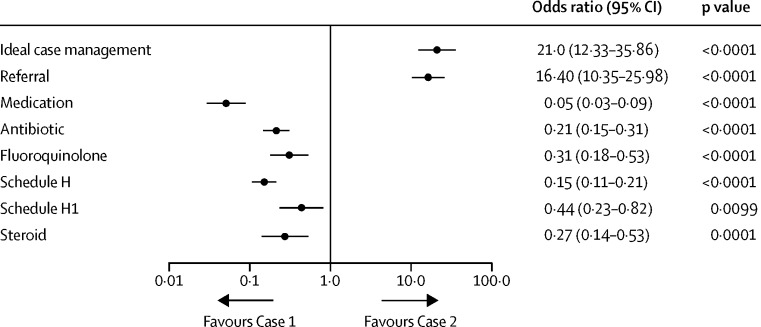
Odds ratios for case management outcomes for Case 1 versus Case 2 Reported odds ratios are from a random-intercepts model using each pharmacy as its own control, with city fixed effects. Odds ratios greater than 1 favour Case 2. Referral is any instance in which the pharmacy staff recommended that the standardised patient seeks further care from a health-care provider. Ideal case management for both cases is defined as a referral without the dispensing of antibiotics or steroids. Schedule H, H1, and X drugs are defined as per the Drugs and Cosmetics Act, 1945, of the Ministry of Health and Family Welfare, Government of India and its amendments.

Figure 1 uses the random-intercept model together with indicator variables for each city to estimate the difference in pharmacy behaviour for the main outcome variables as odds ratios. All these differences were significant and precisely estimated. For instance, the adjusted odds of pharmacies referring a standardised patient with a sputum smear-positive tuberculosis report to a health-care provider without dispensing antibiotics and steroids (ideal case management) was 21·03 (95% CI 12·33–35·86; p<0·0001) for Case 2 relative to Case 1; the odds ratio for antibiotic use was 0·21 (0·15–0·31; p<0·0001) and for fluoroquinolones 0·31 (0·18–0·53; p<0·0001). We also note that of the 497 referrals across the two cases, 301 (60%) were to doctors in the private sector and the remaining 40% were to the public sector (data not shown). In only three instances was the standardised patient referred specifically to a directly observed treatment, short-course (DOTS) centre.

**Figure 2:**
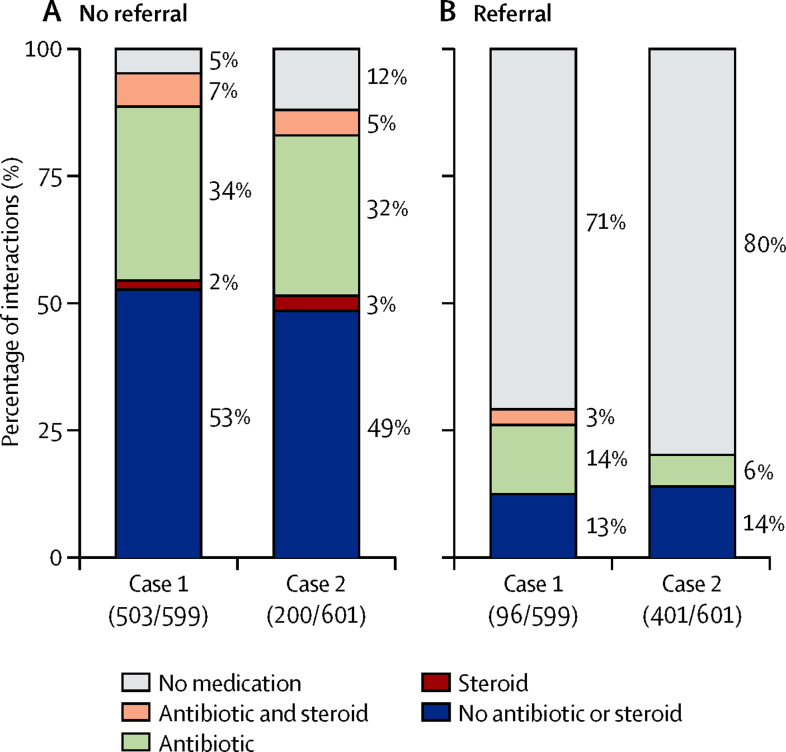
Drug use by referral decisions for two standardised patient cases Each panel describes the use of drugs in each case; the first shows pharmacies that did not refer the standardised patient to another health-care provider (left panel) and the second shows those who did (right panel). Both cases are presented in percentages; the percentages making referral decisions are shown below the case labels in each panel. Percentages indicate the number of interactions within each case-referral category dispensing the indicated types of drugs; percentages may add to more than 100% due to rounding.

In terms of behaviour conditional on referral, the differences between Case 1 and Case 2 reflect, to a substantial degree, the large increase in referrals for Case 2. Figure 2 shows the proportion of interactions that received antibiotics or steroids, or both, or no drug separated by case and referral decision. Both for Case 1 and Case 2, the use of antibiotics or steroids and the total number of drugs fell when the pharmacist referred the patient (0·75 for Case 1, 95% CI 0·48–1·02 *vs* 0·38 for Case 2, 0·29–0·46; data not shown). However, conditioning on the decision to refer, the difference in pharmacist behaviour across the two cases was much smaller.

**Figure 3:**
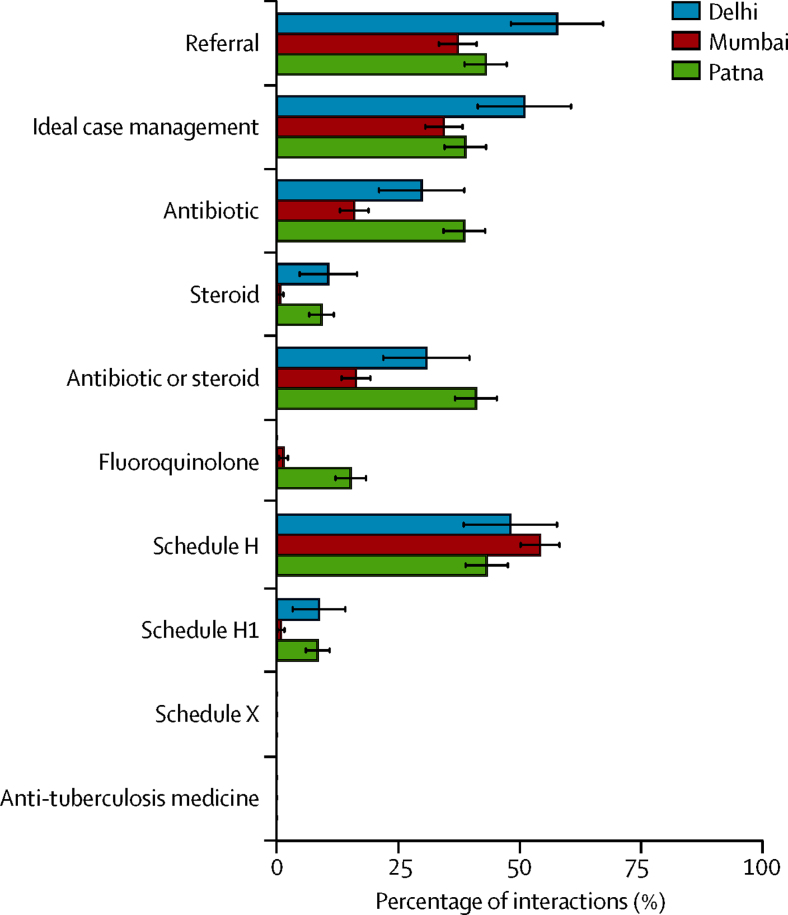
Management of both Case 1 and Case 2 combined by city Referral is any instance in which the pharmacy staff recommended that the standardised patient seek further care from a health-care provider. Ideal case management for both cases is defined as a referral without the dispensing of antibiotics or steroids. Schedule H, H1, and X drugs are defined as per the Drugs and Cosmetics Act, 1945, of the Ministry of Health and Family Welfare, Government of India and its amendments.

The practice of pharmacies varied across cities, although caution is warranted in interpreting these results in view of the different sampling methods used (appendix p 3). We noted similar patterns across the three cities of high use of Schedule H drugs, referrals, and ideal case management (figure 3). Two differences worth highlighting are that compared with Mumbai, the use of antibiotics, steroids, fluoroquinolones, and Schedule H1 drugs were all much higher in Patna; and that there was no fluoroquinolone use in Delhi and little use in Mumbai compared with Patna. These differences are robust to adjustment for differences in the standardised patients used across different cities, an analysis that we did by comparing outcomes only among the (smaller) group of standardised patients who were common to two or more cities (appendix pp 13,14).

**Figure 4:**
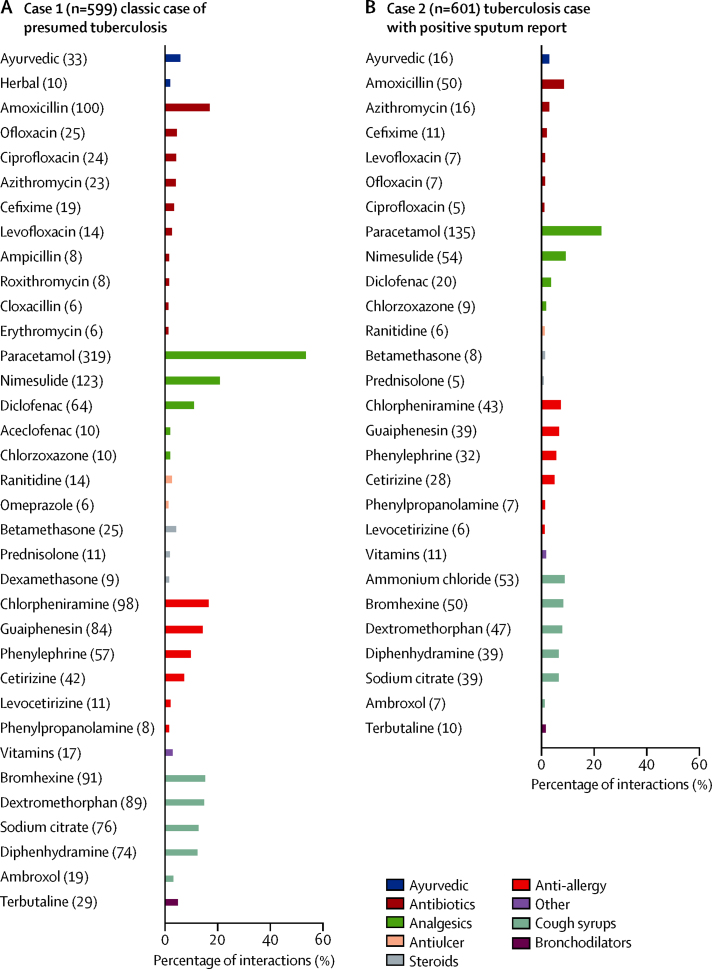
Active ingredients in drugs given for each case The frequency with which each listed active ingredient was contained in drugs given to standardised patients for each case. The number in brackets is the number of interactions in which that active ingredient was recorded.

In terms of type of drugs dispensed, for Case 1, pharmacies dispensed 2·09 drugs on average (95% CI 1·99–2·20; figure 4). The most common classes of drugs dispensed were analgesics such as paracetamol and nimesulide, antibiotics, cough syrups, and anti-allergy drugs. Among antibiotics, amoxicillin was the most common, and 61 (10%) of 599 (95% CI 8–13) pharmacies dispensed fluoroquinolones (eg, ciprofloxacin, levofloxacin, ofloxacin), whereas 45 (8%) of 599 gave steroids such as betamethasone and prednisolone (95% CI 6–10). For Case 2, pharmacies dispensed 0·98 drugs on average (95% CI 0·88–1·09). The classes of drugs dispensed for Case 2 were similar to Case 1, although the overall frequencies were much lower. This finding is again consistent with the result that the difference in behaviour between the two cases was driven, to a large extent, by the sharp increase in referrals for Case 2.

## Discussion

To our knowledge, this is the first study that used standardised patients to examine how pharmacies in India treat patients with tuberculosis symptoms and diagnosed tuberculosis, complementing our recent study that assessed tuberculosis management by health-care providers.^20^ Because the standardised patient method standardises the presentation of the underlying condition across different providers,^23^ the results are reliable, valid, and comparable across pharmacies. The similar patterns we recorded across the three cities suggest that the results might be generalisable to other urban areas in India.

A key finding is that none of the pharmacies in our study dispensed first-line anti-tuberculosis drugs. Concerns regarding the use of anti-tuberculosis drugs by pharmacies seem to be unfounded, at least in major cities, and pharmacies are unlikely sources of irrational drug use that contributes to multidrug-resistant tuberculosis. Why pharmacists do not dispense tuberculosis drugs requires further research, but the fact that tuberculosis drugs (unlike antibiotics such as amoxicillin) are considered toxic and that tuberculosis requires long-term treatment might play a part. Proactiveness of the Indian National Tuberculosis Control Program in including tuberculosis drugs under Schedule H1 and the requirement to document tuberculosis drug prescriptions might also have reduced abuse.

However, our findings showed that 38% of the pharmacies dispensed antibiotics or steroids to people with tuberculosis symptoms but no test results. The use of fluoroquinolones in 7% and steroids in 5% of interactions is especially worrying because these drugs delay tuberculosis diagnosis.^5^, ^24^ Additionally, fluoroquinolones are also an essential part of multidrug-resistant tuberculosis treatment regimens and emerging regimens, so quinolone abuse is a concern.^5^

The widespread use of antibiotics and steroids for respiratory symptoms also has implications for community-acquired infections more generally. Unnecessary use of fluoroquinolones is a major risk factor for creating highly resistant Gram-negative enteric bacteria (eg, extended spectrum beta-lactamase resistance) that might cause diarrhoeal illness, bacteraemia, and other infections, especially in India.^25^ The common use of aminopenicillins (eg, amoxicillin) and macrolides (eg, azithromycin) for respiratory symptoms identified in our study might contribute to resistant strains of common respiratory pathogens such as *Streptococcus pneumoniae* and *Haemophilus influenzae*.^26^ In addition to potentially delaying tuberculosis diagnosis, unnecessary use of steroids is associated with an increased risk of developing lower respiratory tract infection, cellulitis, herpes zoster, and candidiasis.^27^

Our results also clearly show that a first-order problem both in the management of tuberculosis and antimicrobial resistance is the information that patients present to the pharmacist. Confirmed diagnoses discipline what pharmacists do, with sharp increases in ideal management and large decreases in antibiotic use. This dramatic difference suggests that the main challenge faced by pharmacists is confusion about the likely diagnosis, in which case better training regarding tuberculosis symptoms and encouraging early referrals for patient with tuberculosis symptoms might help.

Lastly, our study shows the value of the standardised patient method in tracking inappropriate antibiotic use.^28^ Although prescription audits can be used, prescriptions do not capture the off-prescription use of drugs and often do not include diagnoses.

Although the behaviour change in Case 2 suggests that pharmacists substantially decrease the use of unnecessary drugs when the diagnosis is known, it is unknown why some pharmacists give antibiotics and others do not; neither can we uncover the reasons why pharmacists are unwilling to follow regulations regarding drug use in these three cities. It is unclear whether the variation in our data is explained by the competence and qualification of the person providing advice in pharmacies, which we did not track in the study. Qualitative evidence suggests that a combination of other factors might also be at play, including pharmaceutical industry marketing techniques, business models followed by local providers, and active demand from patients for medicines.^11^, ^29^ Pharmacists in Delhi have described overstock, near-expiry, and undersupply as further factors precipitating misuse of antibiotics and restricted drugs.^11^

Second, we noted significantly higher use of antibiotics and quinolones in Patna than in Mumbai pointing to some differences across cities. We are able to rule out that these differences reflect the composition of standardised patients deployed across cities (appendix p 13), but with an effective sample size of only three cities, we cannot explain this variation. Also, our study does not provide evidence on how pharmacists in rural areas manage patients with tuberculosis or tuberculosis symptoms. Third, our study reflects what happens when pharmacists receive a completely unknown patient as opposed to a known, regular client, or a client who returns to the pharmacist after one round of ineffective treatment. We note though that only 5–6% of pharmacists asked the patient to return (if they did not feel better; appendix p 9). Fourth, differences between Case 1 and Case 2 could reflect variation in the standardised patient profile. Because different standardised patients were assigned to the two cases with no crossover, we cannot assess this possibility. Generally, the inclusion of standardised patient characteristics has little effect on estimated coefficients in previous standardised patient studies and our coefficients remain stable when we account for standardised patient sex, height, and weight (appendix pp 13,14).

To conclude, our study adds to the growing evidence in India on antibiotic abuse, but also underscores that the use of antibiotics is mediated by drug category and the information that patients present. Although antibiotic use is high and such use can delay diagnosis, none of the pharmacies dispensed anti-tuberculosis drugs and the use of stronger fluoroquinolone antibiotics and heavily restricted drug classes was low. Furthermore, the use of all antibiotics decreased sharply when the patient's diagnosis was revealed to the pharmacists. These findings can inform interventions to engage pharmacies in tuberculosis control and antimicrobial stewardship.

## Figures and Tables

**Table 1:** Standardised patient case descriptions

	**Case description**	**Presentation of standardised patient**	**Expected case management**
Case 1	Classic case of presumed tuberculosis with 2–3 weeks of cough and fever and directly seeking care from a chemist or pharmacist	Case 1 presents with the opening statement, “Sir, I have cough and fever that is not getting better. Please give me some medicine.” At presentation, this case has had a 2–3 week cough, which occurred more during early morning and night, accompanied by a 2–3 week, on-and-off, low-grade fever. The patient was producing sputum that did not contain any blood. The case would admit to a loss of appetite and to his or her clothes becoming a bit loose if prompted by the chemist. If the chemist asked about taking medicines for this illness, the patient would say no	Verbal or written referral to a DOTS centre or a health-care provider without dispensing any antibiotics (including anti-tuberculosis drugs and fluoroquinolones) or steroids
Case 2	Chronic cough with a positive sputum smear report for tuberculosis from a government dispensary and directly seeking care from a chemist or pharmacist	Case 2 presents with a positive sputum smear result visiting a chemist, presenting with the opening statement, “Sir, I am having cough for nearly a month now and also have fever.” While showing a positive sputum report to the chemist, the patient continues, “I went to the government dispensary and they asked me to get my sputum tested. I have this report. Can you please give me some medicine?” At presentation, this case has had a cough for 1 month and produces sputum without blood, accompanied by a 1 month, on-and-off, low-grade fever, which was more during evening times. Similar to Case 1, the case would admit to a loss of appetite and to his or her clothes becoming a bit loose if prompted by the chemist. If the chemist asked about taking medicines for this illness, the patient would say no	Verbal or written referral to a DOTS centre or a health-care provider without dispensing any antibiotics (including anti-tuberculosis drugs and fluoroquinolones) or steroids

DOTS=directly observed treatment, short-course.

**Table 2:** Management of Case 1 and Case 2 for all cities and for Patna and Mumbai only

		**All cities (Delhi, Mumbai, and Patna)**		**Patna and Mumbai only**	
		Case 1	Case 2	Case 1	Case 2
Number of interactions		599	601	548	548
Referral		96, 0·16 (0·13–0·19)	401, 0·67 (0·63–0·70)	75, 0·14 (0·11–0·17)	362, 0·66 (0·62–0·70)
Ideal case management		80, 0·13 (0·11–0·16)	372, 0·62 (0·58–0·66)	64, 0·12 (0·09–0·14)	335, 0·61 (0·57–0·65)
Drugs					
	Number of drugs	2·09 (1·99–2·20)	0·98 (0·88–1·09)	2·07 (1·97–2·18)	0·97 (0·86–1·08)
	Antibiotic	221, 0·37 (0·33–0·41)	98, 0·16 (0·13–0·19)	200, 0·36 (0·32–0·41)	88, 0·16 (0·13–0·19)
	Steroid	45, 0·08 (0·05–0·10)	16, 0·03 (0·01–0·04)	37, 0·07 (0·05–0·09)	13, 0·02 (0·01–0·04)
	Antibiotic or steroid	230, 0·38 (0·34–0·42)	104, 0·17 (0·14–0·20)	208, 0·38 (0·34–0·42)	94, 0·17 (0·14–0·20)
	Fluoroquinolone	61, 0·10 (0·08–0·13)	23, 0·04 (0·02–0·05)	61, 0·11 (0·08–0·14)	23, 0·04 (0·03–0·06)
	Schedule H	401, 0·67 (0·63–0·71)	188, 0·31 (0·28–0·35)	367, 0·67 (0·63–0·71)	172, 0·31 (0·27–0·35)
	Schedule H1	37, 0·06 (0·04–0·08)	19, 0·03 (0·02–0·05)	31, 0·06 (0·04–0·08)	16, 0·03 (0·02–0·04)
	Schedule X	0	0	0	0
	Anti-tuberculosis	0	0	0	0

Data are n, proportion (95% CI) or mean (95% CI).
