# Supplementary material for: Use of standardised patients to assess antibiotic dispensing for tuberculosis by pharmacies in urban India: a cross-sectional study
Source: Lancet Infect Dis. 2016 Nov;16(11):1261–8. doi: 10.1016/S1473-3099(16)30215-8 (PMC5067371; doi:10.1016/S1473-3099(16)30215-8)
Supplement: Supplementary appendix [file mmc1.pdf]

THE LANCET Infectious Diseases

Supplementary webappendix

This webappendix formed part of the original submission and has been peer reviewed. We post it as supplied by the authors.

Supplement to: Satyanarayana S, Kwan A, Daniels B, et al. Use of standardised patients to assess antibiotic dispensing for tuberculosis by pharmacies in urban India: a cross-sectional study. *Lancet Infect Dis* 2016; published online Aug 24. http://dx.doi.org/10.1016/S1473-3099(16)30215-8.

SUPPLEMENTARY APPENDIX

Supplement to:

Use of standardised patients to assess antibiotic dispensing for tuberculosis by pharmacies in urban India: a cross-sectional study

Srinath Satyanarayana, Ada Kwan, Benjamin Daniels, Ramnath Subbaraman, Andrew McDowell, Sofi Bergkvist, Ranendra K. Das, Veena Das, Jishnu Das, Madhukar Pai

Corresponding author

Prof. Madhukar Pai, MD, PhD

Director, McGill Global Health Programs

Associate Director, McGill International TB Centre

Dept of Epidemiology & Biostatistics

1020 Pine Ave West

Montreal, QC H3A 1A2, Canada

Tel: 514-398-5422

Fax: 514-398-4503

Email: madhukar.pai@mcgill.ca

Supplementary Methods

1. Process of selecting the pharmacies

Appendix Table A1 summarizes pharmacy recruitment and study implementation across the three cities. In Delhi, 54 chemists from 28 low-income localities were convenience sampled as a pilot study for methodological validation and power calculations. In urban Patna (defined as Patna, Danapur, and Phulwarisharif blocks) and 15 high-slum population wards of Mumbai, a lane-by-lane mapping exercise conducted between January and August 2014 served as a complete list of pharmacies that were operating in these areas at the time.

Additionally, during the data collection period for this study in Mumbai and Patna, urban TB programs implemented by Private-Provider Interface Agencies (PPIA) were recruiting and enrolling pharmacist or pharmacist assistants into TB referral and treatment networks in both Mumbai and Patna. At the time of sampling for our study in these two cities, we decided to stratify our sample by PPIA program enrollment. The description of the program serves to support sampling weights (Appendix Table A2) applied to achieve the urban area estimates for Mumbai and Patna but stratified findings based on PPIA program enrollment are not presented in this paper. From the mapping lists in Mumbai and Patna, we obtained lists of enrolled pharmacies in the PPIA program (in Patna as of September 25, 2014 for round 1 and as of September 15, 2015 for round 2; in Mumbai as of January 30, 2015 for round 1 and as of September 30, 2015 for round 2) and matched them back to the complete mapping lists to obtain a sampling universe stratified by PPIA enrollment status.

From these lists, in urban Patna, we randomly selected 125 pharmacies from the 268 pharmacies enrolled into the PPIA program and 125 from the 602 pharmacies not enrolled in the program across the two sampling rounds. The geographical frame covered all 40 wards in Danapur block, all 28 wards in Phulwari Shariff block, and 34 wards selected in collaboration with the PPIA out of 73 wards in Patna block. For both of the random samples in Patna, we provided a reserve list, which could replace originally sampled pharmacists found to be permanently closed at the time of data collection for the purposes of surveillance. One pharmacist became enrolled in the PPIA program between the data collection rounds and 10 others were closed and replaced by an identically sampled replacement in the second round.

In Mumbai, four of the 15 high-slum population wards were purposively selected for this study in collaboration with the PPIA to reflect different geographical areas with both registered and unregistered slums and accessible transit for our field team. These four wards had a total of 1,160 pharmacies covering a total population of 3,181,264, of which 2,275,555 people (72%) were living in an area identified as a slum.[1,2] In the same four wards, we assigned SP visits to all chemists enrolled into the PPIA program (48 as of September 2015) and then randomly selected 250 chemists from 1,094 chemists who were not enrolled into the urban TB program in the four wards across the two sampling rounds. This included three

chemists who became enrolled in the PPIA program between rounds 1 and 2, as well as 10 that were closed and replaced by an identically sampled replacement in the second round. We used Stata Version 13 (Stata Corp, College Station, TX) to generate the random samples in Patna and Mumbai.

Appendix Table A1. Study Design by City

(1) Period	(2) Data collection dates	(3) Chemist recruitment	(4) Number of standardized patients	(5) Interaction details
**Delhi (Pilot)**	April 1–23, 2014	Convenience sample for pilot	9 unique SPs	51 Case 1 interactions<br>53 Case 2 interactions
**Delhi Total**			9 unique SPs	54 unique chemists
**Patna Round 1**	November 5–12, 2014	Random sampling of chemists enrolled in an urban TB program in urban Patna (enrollment date of September 25, 2014), and random sampling of chemists not enrolled in an urban TB program (all Danapur block, all Phulwarisharif block, and purposively selected areas in Patna block).	9 unique SPs	122 Case 1 interactions
**Patna Round 2**	November 19–29, 2015	Identical random sampling to extend Case 1 sample (enrollment date of September 15, 2015). Case 2 sent to all chemists now in Case 1 sample. Identically sampled replacement used if Round 1 chemist unavailable in Round 2.	8 unique SPs	128 Case 1 interactions<br>250 Case 2 interactions
**Patna Total**			12 unique SPs	260 unique chemists
**Mumbai Round 1**	April 1–11, 2015	Random sample for chemists not enrolled in an urban TB program and a census of all chemists enrolled in the program in four wards (enrolment date of January 30, 2015)	13 unique SPs	169 Case 1 interactions
**Mumbai Round 2**	October 7 – November 3, 2015	Identical random sampling and census (enrolled date as of September 30, 2015), to extend Case 1 sample. Case 2 sent to all chemists now in Case 1 sample. Identically sampled replacement used if Round 1 chemist unavailable in Round 2.	8 unique SPs	129 Case 1 interactions<br>298 Case 2 interactions
**Mumbai Total**			14 unique SPs	308 unique chemists

Appendix Table A2. Pharmacies Eligible for Sampling and Observation Weighting by City

(1) City	(2) Sample	(3) Pharmacies	(4) Interactions	(5) City-Sample Observation Weight		(6)
**Delhi**	**Full Sample**	n/a	Case 1: 51<br>Case 2: 53		=	1.00
**Mumbai**	**PPIA**	48	Case 1: 45<br>Case 2: 48	(48/1142)/(48/298)	=	0.261
**Mumbai**	**Non-PPIA**	1094	Case 1: 253<br>Case 2: 250	(1094/1142)/(250/298)	=	1.142
**Patna**	**PPIA**	268	Case 1: 125<br>Case 2: 126	(268/870)/(126/250)	=	0.611
**Patna**	**Non-PPIA**	602	Case 1: 125<br>Case 2: 124	(602/870)/(124/250)	=	1.395

1.1 Deviations from Sampling Protocol

We had anticipated the possibility of being unable to reach all our sampled pharmacies in our design by oversampling and selecting a random subset of pharmacies to be held in reserve for the samples in each city. In practice after our pilot in Delhi, the SPs were sent to conduct the interaction at the

sampled pharmacist at a given location up to two times, and if the interaction could not be successfully completed in two visits (e.g., the pharmacist had closed his shop), the originally sampled pharmacist was dropped and replaced with a reserve.

Given a total of 622 pharmacists, we should have had 1244 interactions, since each pharmacist was assigned two cases and one interaction per case. Since pharmacists were assigned two cases, reserves were pulled in for the remaining case if one had already been successfully completed (e.g., in the previous round). Since our reserves were used at the interaction rather than the pharmacy level, we could have situations where Case 1 was completed with one pharmacy, which then shut down or moved and Case 2 had to be completed with a reserve. We consider the 44 unpaired visits where 1 case was completed to be "missing" their paired visit to the same pharmacy.

Across cities, 1 pharmacist from the convenience sample in Delhi received Case 1 but not Case 2 and 3 received Case 2 but not Case 1. In Patna and Mumbai, there were 20 originally sampled chemists who successfully received Case 1 but did not receive Case 2. For each of these instances, a reserve was used as a replacement for the Case 2 interaction through an identical sampling strategy. This resulted in 20 pharmacy reserve pharmacists who received Case 2 only (10 in Mumbai and 10 in Patna), resulting in 40 unmatched interactions. In Patna and Mumbai, reserves were used either because (i) the sampled pharmacy shop was permanently closed or had moved to another location, or (ii) the pharmacist was not available during the data collection period. See Appendix Figure A1.

Appendix Figure A1. Pharmacist sampling and visit completion

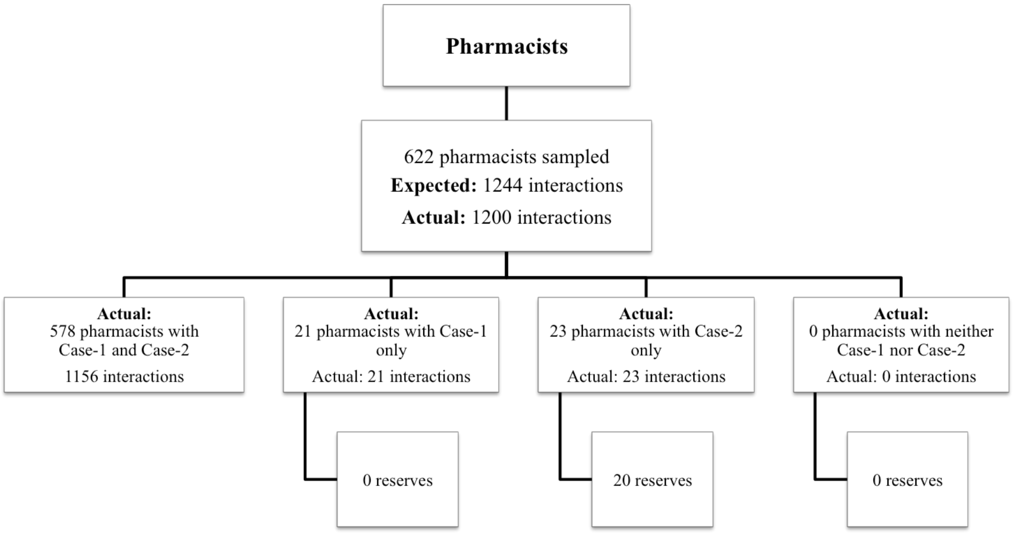

2. Standardized Patient Cases and Recruitment

We have previously described the validation of the SP methodology for presentations of tuberculosis.[3] We demonstrated that (1) participation in the study had minimal to no risk for the SPs or health care providers, (2) the likelihood of SP detection among visited providers was 5%, which was very low (and lower than other studies), confirming that SPs were considered real by health providers who were visited, and (3) the abilities of the SPs to recall what occurred during the interaction was strongly correlated with what actually happened. Additionally, because the SPs pay the fees requested by the healthcare provider, there is no loss to provider income from participation in the study.

Simultaneously with the interactions published in the Delhi pilot study, the two cases in this paper were designed and piloted in Delhi for presentation at pharmacies. After the pilot in Delhi, the cases were adjusted for the Patna and Mumbai contexts.

For each case, both the clinical case presentation and social contexts were developed and agreed upon by a technical advisory group, which included international and national TB experts and clinicians. Clinical aspects were standardized across the three cities, and the scripts were adjusted to account for different social and family contexts across cities. Script development occurred under the guidance of an anthropologist (VD) and with the support of supervisors and participating SP recruits. Scripts were in English and Hindi for the three cities and additionally translated into Marathi for Mumbai.

A different cohort of SPs, in apparently healthy condition, was recruited and trained in each city; some of the SPs participated in data collection in more than one city. The 24 individuals (7 females and 17 males) hired as SPs in total included both new recruits and individuals who had participated in previous SP studies assessing other health conditions. The SPs, although recruited specifically to fit each case, differed in age, gender, height, and weight. The average age of all the SPs was 30. The youngest was 21, and the oldest was 39. The 17 males weighed 50 to 74 kilograms and were 160 to 184 centimeters tall. The 7 females weighed 46 to 72 kilograms and were 147 to 160 centimeters tall.

In each round of data collection and in each city, SPs were assigned to either Case 1 or Case 2 and never to both cases in order to avoid detection, since each sampled chemist was assigned to receive both cases. Further details on the cohort for each city were as follows:

- In Delhi, a total of nine individuals were recruited and trained for three weeks. Five individuals (three female) were trained as Case 1, and four different individuals (one female) were trained as Case 2.
- In Patna, a total of 12 individuals participated as SPs. During the first round, nine individuals (four female) were trained for 5 days (October 27 – November 4, 2014) to depict Case 1. Then

during the second round, eight individuals were rehired and received two days of refresher training (November 17 - 18, 2015).

- In Mumbai, a total of 14 individuals participated as SPs. During the first round, 13 individuals (two female) were trained for 10 days (March 20 – 31, 2015) to depict Case 1. During the second round, eight individuals were rehired and received two days of refresher training (October 5 - 6, 2015).

The training of SPs ensured that they (a) correctly presented the cases, (b) correctly recalled the interaction with the pharmacy staff, and (c) avoided detection. The first two aims were achieved through classroom training in case presentation and testing of recall, as well as mock interviews and dry runs that were supervised in the field.

For the third aim, SPs were taught to avoid detection by the following methods. First, our recruitment strategy ensured that SPs came from low-income areas or slums from the same cities in which the project was located, and the areas from which they came were far from the field sites. This meant that their clothing, mannerisms, and speech were very close to the ordinary patients who visited pharmacists, but they would not have been personally known in the study areas. Second, previous observations in pharmacies and chemist shops were conducted by supervisors in order to observe the patterns of interaction (e.g., mode of address), and we ensured that SPs approximated those patterns of interaction. Third, during the training, SPs were taught to internalize completely the characters and the details of their mock stories through which the character was made alive to them. In mock interviews during training, supervisors would add unscripted questions with regard to family or neighborhood that SPs could answer spontaneously because they were of the actual social background that was being approximated in the characters they were portraying. Finally, dry runs were conducted in which the supervisor was present in the shop on the pretense of buying something such as toothpaste or an over-the-counter cough syrup and thus could watch the interaction and offer corrections later.

The different number of training days across cities and rounds of data collection was determined by how many of the individuals in the cohort were new recruits and among the experienced SPs, how recent their last experience was depicting the case they were assigned. For example, some SPs who participated in Patna data collection as Case 1 had worked with us in the Delhi pilot, and a briefer, refresher training was conducted.

3. Identification of drugs given by pharmacists

In order to assess drug use, all labelled medicines prescribed by the pharmacies were digitized and stored and then coded by two doctors with expertise in TB (SS) and infectious diseases (RS). Blinded from any

provider identifying details, they identified and categorized medicines as steroids, anti-TB drugs, fluoroquinolones, or other broad-spectrum antibiotics under maker-checker procedure. They also identified whether the individual drug is listed under Schedule H, Schedule H1, or Schedule X of India's Ministry of Health and Family Welfare's Drugs and Cosmetics Rules Act, 1945,[4] or its amendments. Discrepancies in categorization between the two coders were resolved by consensus.

It is important to mention that Schedule H drugs also include common prescription-only drugs such as Ibuprofen and Cetirizine. Similarly, some but not all fluoroquinolones are listed on Schedule H1. For instance, Ciproflaxacin and Ofloxacin remain in Schedule H, but Levofloxacin, Moxifloxacin, Prulifloxacin and Sparfloxacin are Schedule H1 drugs. Finally, loose or unlabeled pills were dispensed in 28 of 1200 interactions, and we made no further attempts to identify them.

4. Sample size calculations

Appendix Table A3 shows our sample size calculations for various assumptions of interaction outcome frequencies, which we calibrated against the results from the Delhi pilot sample. Based on the results of the pilot study in Delhi a sample size of 250 pharmacies per city would allow us to estimate the proportion of ideal case management for each case with a precision of +/-5%. In Delhi, ideal case management was 31% for Case 1 and 70% for Case 2 (under "Ideal Case Management" in Table 2, Columns (1) and (2)). As seen from Column (3) in Table A3, the sample size required for an outcome proportion of 30% is 252 in Mumbai and 236 in Patna. As the computation is symmetric around 50%, this is the same sample size required for an outcome proportion of 70% as well. Note that we did not account for potential design effects as we chose equal probability samples from the entire list frame, as opposed to a clustered random sample. Given that this is a binary outcome variable, the specific formula is:

$$n = [Np(1-p)]/ [(d^2/Z^2*(N-1)+p*(1-p)]$$

where:
n = Required sample size per SP city-case set
N = Population size
p = Hypothesized outcome proportion in the population
d = Absolute confidence limits (as % of 100)
Z = Z-score (for 95% Confidence levels Z-score=1.96).[5]

Appendix Table A3. Sample Size Calculations

	Binary outcome frequency scenarios				
	**(1)**	**(2)**	**(3)**	**(4)**	**(5)**
		**Mumbai**			
**Pharmacies in sampling frame**	1,142	1,142	1,142	1,142	1,142
**Hypothetical outcome proportion**	10%	20%	30%	40%	50%
**Width of confidence interval**	+/-5%	+/-5%	+/-5%	+/-5%	+/-5%
**Confidence level**	95%	95%	95%	95%	95%
**Required sample size**	**124**	**203**	**252**	**279**	**288**
		**Patna**			
**Pharmacies in sampling frame**	870	870	870	870	870
**Hypothetical outcome proportion**	10%	20%	30%	40%	50%
**Width of confidence interval**	+/-5%	+/-5%	+/-5%	+/-5%	+/-5%
**Confidence level**	95%	95%	95%	95%	95%
**Required sample size**	**120**	**192**	**236**	**260**	**267**

5. Clinical outcomes by city and case

In the main text we provide key outcome variables for all cities and for Patna and Mumbai only, for both cases combined. To accompany the description in the text, Appendix Table A4 below provides the full set of outcome variables for each city and case.

Appendix Table A4. Clinical outcomes for Case 1 and 2 combined across cities and by city.

	(1)<br>All Case 1	(2)<br>All Case 2	(3)<br>Delhi Case 1	(4)<br>Delhi Case 2	(5)<br>Mumbai Case 1	(6)<br>Mumbai Case 2	(7)<br>Patna Case 1	(8)<br>Patna Case 2
Number of Interactions	***599***	***601***	***51***	***53***	***298***	***298***	***250***	***250***
**Referral**	96/599	401/601	21/51	39/53	36/298	186/298	39/250	176/250
	0.16 [0.13–0.19]	0.67 [0.63–0.70]	0.41 [0.28–0.55]	0.74 [0.62–0.85]	0.11 [0.07–0.15]	0.61 [0.55–0.67]	0.15 [0.10–0.19]	0.70 [0.64–0.76]
**Ideal Case Management**	80/599	372/601	16/51	37/53	31/298	174/298	33/250	161/250
	0.13 [0.11–0.16]	0.62 [0.58–0.66]	0.31 [0.19–0.44]	0.70 [0.57–0.82]	0.09 [0.06–0.13]	0.58 [0.52–0.64]	0.12 [0.08–0.16]	0.64 [0.57–0.70]
**Medications**								
**Antibiotic**	221/599	98/601	21/51	10/53	57/298	38/298	143/250	50/250
	0.37 [0.33–0.41]	0.16 [0.13–0.19]	0.41 [0.28–0.55]	0.19 [0.08–0.29]	0.18 [0.14–0.23]	0.12 [0.08–0.15]	0.58 [0.51–0.65]	0.20 [0.14–0.25]
**Steroid**	45/599	16/601	8/51	3/53	3/298	1/298	34/250	12/250
	0.08 [0.05–0.10]	0.03 [0.01–0.04]	0.16 [0.06–0.26]	0.06 [0–0.12]	0.01 [0–0.02]	0 [0–0.01]	0.14 [0.09–0.18]	0.05 [0.02–0.08]
**Antibiotic or Steroid**	230/599	104/601	22/51	10/53	58/298	39/298	150/250	55/250
	0.38 [0.34–0.42]	0.17 [0.14–0.20]	0.43 [0.30–0.57]	0.19 [0.08–0.29]	0.19 [0.14–0.23]	0.12 [0.08–0.16]	0.60 [0.54–0.67]	0.22 [0.16–0.27]
**Mentioned TB in Interaction**	12/599	118/601	1/51	14/53	4/298	47/298	7/250	57/250
	0.02 [0.01–0.03]	0.20 [0.16–0.23]	0.02 [0–0.06]	0.26 [0.15–0.38]	0.01 [0–0.03]	0.16 [0.12–0.20]	0.03 [0.01–0.05]	0.22 [0.17–0.28]
**Fluoroquinolone**	61/599	23/601	0/51	0/53	5/298	3/298	56/250	20/250
	0.10 [0.08–0.13]	0.04 [0.02–0.05]	0 [–]	0 [–]	0.02 [0–0.03]	0.01 [0–0.02]	0.22 [0.17–0.28]	0.09 [0.05–0.13]
**Schedule H**	401/599	188/601	34/51	16/53	207/298	116/298	160/250	56/250
	0.67 [0.63–0.71]	0.31 [0.28–0.35]	0.67 [0.54–0.80]	0.30 [0.18–0.43]	0.70 [0.64–0.75]	0.38 [0.32–0.44]	0.65 [0.59–0.72]	0.23 [0.17–0.28]
**Schedule H1**	37/599	19/601	6/51	3/53	2/298	3/298	29/250	13/250
	0.06 [0.04–0.08]	0.03 [0.02–0.05]	0.12 [0.03–0.21]	0.06 [0–0.12]	0.01 [0–0.02]	0.01 [0–0.01]	0.11 [0.07–0.15]	0.05 [0.02–0.08]
**Schedule X**	0/599	0/601	0/51	0/53	0/298	0/298	0/250	0/250
	0 [–]	0 [–]	0 [–]	0 [–]	0 [–]	0 [–]	0 [–]	0 [–]
**Anti-Tuberculosis**	0/599	0/601	0/51	0/53	0/298	0/298	0/250	0/250
	0 [–]	0 [–]	0 [–]	0 [–]	0 [–]	0 [–]	0 [–]	0 [–]
**Interaction Statistics**								
**Asked SP to Return**	34/595	30/594	3/51	7/53	1/296	1/291	30/248	22/250
	0.06 [0.04–0.08]	0.05 [0.03–0.07]	0.06 [0–0.12]	0.13 [0.04–0.22]	0 [0–0.01]	0 [0–0.01]	0.12 [0.08–0.17]	0.10 [0.06–0.14]
**Duration of Interaction (min)**	1.73	1.62			1.37	1.49	2.27	1.80
	[1.60–1.86]	[1.49–1.76]			[1.25–1.49]	[1.41–1.57]	[1.98–2.55]	[1.46–2.14]
**Number of Questions Asked**	1.44	1.20	1.27	1.06	1.06	1.07	1.99	1.41
	[1.30–1.57]	[1.09–1.31]	[0.92–1.63]	[0.77–1.34]	[0.86–1.25]	[0.92–1.22]	[1.75–2.23]	[1.18–1.63]
**Price (INR)**	61.02	44.15	47.45	36.70	50.70	58.56	77.92	40.23
	[56.30–65.75]	[38.23–50.07]	[34.71–60.20]	[19.98–53.42]	[46.00–55.39]	[49.83–67.30]	[68.88–86.96]	[30.01–50.45]
**Price (USD)**	0.98	0.71	0.76	0.59	0.81	0.94	1.25	0.64
	[0.90–1.05]	[0.61–0.80]	[0.56–0.96]	[0.32–0.85]	[0.74–0.89]	[0.80–1.08]	[1.10–1.39]	[0.48–0.81]
**Number of Lab Tests Ordered**	0.01	0.01	0.02	0.00	0.01	0.02	0.00	0.01
	[0.00–0.02]	[0.00–0.02]	[0–0.06]	[–]	[0–0.03]	[0.00–0.04]	[–]	[0.00–0.02]
**Any Medication Given**	507/599	257/601	38/51	20/53	267/298	149/298	202/250	88/250
	0.85 [0.82–0.88]	0.43 [0.39–0.47]	0.75 [0.63–0.86]	0.38 [0.25–0.51]	0.91 [0.88–0.94]	0.50 [0.44–0.56]	0.83 [0.78–0.88]	0.36 [0.30–0.42]
**Number of Medicines**	2.09	0.98	2.29	1.15	1.79	0.96	2.51	1.00
	[1.99–2.20]	[0.88–1.09]	[1.81–2.78]	[0.69–1.61]	[1.67–1.90]	[0.82–1.10]	[2.32–2.69]	[0.80–1.20]

Notes: Data are either mean and 95% confidence interval for continuous variables; or observed proportion, estimated population proportion, and 95% confidence interval for binary variables. Referral indicates any situation in which the chemist recommended the SP to visit a health care provider. Ideal case management is defined as a referral without the dispensing of antibiotics or steroids. USD prices calculated using the 1 Aug 2015 exchange rate of 0.016USD/INR. Schedule H, H1 and X medications are defined as per the Drugs and Cosmetics Act, 1945, of the Ministry of Health and Family Welfare, Government of India and its amendments.

6. Model for estimating the odds ratios of management behaviours for the two cases at each pharmacy

Using an econometric model, we are interested in estimating the differences in case management and medication use across a patient with presumptive TB (Case 1) and a patient with confirmed TB (Case 2), to determine the extent to which the behaviors of pharmacies change based on the confirmation of the diagnosis. We utilize the following variables:

Y_{ij} = Outcome (1 = Yes, 0 = No) for pharmacist *i* in SP case *j,* where Outcome can be ideal case management, antibiotic use, or any other binary outcome variable;

C_{ij} = Case Exposure (Case 1 = 0; Case 2 =1) for pharmacist *i* in SP case *j*;

L_i = Study location for pharmacy *i* (Delhi = 1, Mumbai = 2, Patna = 3)

Suppose we are first interested in estimating the marginal effects of Case Exposure on ideal case management. For marginal effects, linear models like OLS are consistent for binary variables, but not efficient. Nevertheless, they require fewer assumptions on the structure of the error term and are therefore robust to misspecification in the functional form of the error term. Suppose that every pharmacy has an unobserved ability level, v_i, such that pharmacies with higher v_i are also more likely to correctly manage the patient. Therefore,

$$Y_{ij} = a + b.C_{ij} + c.L_i + v_i + e_{ij}$$

If we were to observe real patients, it may be the case that patients who do not know their diagnosis choose a higher v_i pharmacy. Therefore, the choice of the pharmacy by the patient confounds the estimated marginal effect we are interested in. However, with two SP visits to each pharmacy, one for each case, the difference in Y_{i1} and Y_{i2} yields a consistent estimate of b, purged of v_i. Note that the simple linear OLS model can be estimated either through pairwise differences, i.e., by subtracting each pharmacy's performance in Case 1 from its performance in Case 2, or using the conditional expectation function, and noting that because every pharmacy receives both cases, the $E(v_i \mid \text{Case 1}) = E(v_i \mid \text{Case 2})$. This is the difference between OLS with and without fixed-effects at the level of the pharmacy. Finally, the same argument will hold for why the OLS with random intercepts, which assumes that $Corr(v_i, C_{ij}) = 0$ will yield the same coefficients. Specifically, because there is no active choice and both pharmacies received two cases each, correlations between the pharmacy-specific intercept and the choice of cases are ruled out by design. In practice, there will be two differences between the marginal effects estimated through OLS, OLS with fixed-effects and OLS with random intercepts:

- Precision will be higher with the random intercepts model, as (a) there are fewer degrees of freedom in the fixed-effects model (a fixed-effect is estimated for each pharmacy) and (b) the OLS does not take into account the specific error structure.
- In practice, the actual estimates may also differ because not every case was completed with every pharmacist. For instance, if low-ability pharmacies are more likely to close and Case 1 was attempted first, we may have more Case 1 interactions with higher ability pharmacists. Similarly, the inclusion of city-level indicator variables helps eliminate any potential effects arising from differences in the number of cases of each type across cities.

Estimates of odds-ratios using a logit error structure may differ because the non-linearity in the logit function implies that the logic of the conditional expectation function no longer holds. That is, since $E(F(v_i \mid \text{Case 1})$ is not $F(E(v_i \mid \text{Case 1})$, additional differences may arise between the logit model, the logit model with fixed-effects and the logit model with random intercepts. In practice, given that most of our outcome variables have observed proportions between 20% and 80%, these differences should be small since the logit function is close to linear in this range.

In our econometric model, we fit a random intercept logistic regression model to estimate the differences between the management of Case 1 and Case 2, producing odds-ratios for Case 2 : Case 1. The random intercepts logit model is illustrated below with the following variables: v_{0i} = Random intercept for pharmacy i in [1 , 622] and

$$\text{Logit}(Y_{ij}) = \beta_0 + v_{0i} + \beta_2 * C_{ij} + \beta_3 * L_i + \varepsilon_{ij}$$

where the exponentiation to the 'e' of the coefficient β_2 is interpreted as the odds ratio for optimal management of Case 2 against Case 1 by each pharmacy. The random intercepts v_{0i} are distributed ~N (0, σ_p^2), and ε_{ij} is distributed as a standard logistic distribution.

Appendix Table A5 first shows the proportions for the outcome variables we consider for each of the two cases. The odds-ratios presented in Column (7) reflect estimates from a logit model *without* random intercepts, fixed-effects or city-level indicator variables.

Appendix Table A5. Summary of differences between Case 1 and Case 2 - No Controls Logit Model

Variable	(1) Case 1	(2) N	(3) Proportion	(4) Case 2	(5) N	(6) Proportion	(7) Odds Ratio	(8) 95% Lower Bound	(9) 95% Upper Bound	(10) P-Value
**Ideal Case Management**	80	599	0.13	372	601	0.62	10.54	7.91	14.05	0.0000
**Referral**	96	599	0.16	401	601	0.67	10.51	7.97	13.85	0.0000
**Medication**	507	599	0.85	257	601	0.43	0.14	0.10	0.18	0.0000
**Antibiotic**	221	599	0.37	98	601	0.16	0.33	0.25	0.44	0.0000
**Fluoroquinolone**	61	599	0.10	23	601	0.04	0.35	0.21	0.58	0.0000
**Schedule H**	401	599	0.67	188	601	0.31	0.22	0.18	0.29	0.0000
**Schedule H1**	37	599	0.06	19	601	0.03	0.50	0.28	0.87	0.0150
**Steroid**	45	599	0.08	16	601	0.03	0.34	0.19	0.60	0.0002

Appendix Table A6 then shows estimates of the marginal effects using linear models that include city fixed effects (Columns 1-3) and from non-linear logit models that include city fixed effects (Columns 4-6). (For all regression coefficients, *** = p<0.01, ** = p<0.05, * = p<0.1.) For both types of models, we show the base estimates, pharmacy fixed-effects estimates, and pharmacy random-intercepts estimates. We also report the p-value from a Hausman test comparing the fixed-effects and the random-intercepts coefficients from Columns (5) and (6). When this test does not return a significant result, it indicates that the random-intercepts assumption is likely to be fulfilled.

Appendix Table A6. Summary of differences between Case 1 and Case 2 under various city-fixed-effects models

	(1)	(2)	(3)	(4)	(5)	(6)	(7)	(8)	(9)
	Linear Differences			Odds Ratios				Proportions	
	OLS	OLS Fixed Effects	OLS Random Intercepts	Logit	Logit Fixed Effects	Logit Random Intercepts	Hausman Test P-value	Case 1	Case 2
**Ideal Case Management**	0.485***	0.488***	0.485***	10.876***	21.143***	21.031***	0.838	0.134	0.619
**Referral**	0.506***	0.509***	0.507***	11.047***	16.474***	16.398***	0.863	0.160	0.667
**Medication**	-0.419***	-0.420***	-0.419***	0.130***	0.051***	0.051***	0.772	0.846	0.428
**Antibiotic**	-0.206***	-0.202***	-0.205***	0.309***	0.220***	0.214***	0.193	0.369	0.163
**Fluoroquinolone**	-0.063***	-0.057***	-0.063***	0.325***	0.340***	0.309***	0.030	0.102	0.038
**Schedule H**	-0.357***	-0.353***	-0.356***	0.220***	0.154***	0.151***	0.397	0.669	0.313
**Schedule H1**	-0.030**	-0.028**	-0.030***	0.483**	0.467**	0.437***	0.150	0.062	0.032
**Steroid**	-0.049***	-0.045***	-0.048***	0.321***	0.297***	0.274***	0.072	0.075	0.027

As Table A6 shows, all marginal effects are identical whether estimated through OLS, fixed-effects or random-intercepts models. For odds ratios, the fixed-effects and random-intercepts models are again identical for all outcome variables except fluoroquinolone use, but even there the absolute difference between the coefficients is small. However, the fixed-effects and random intercepts models differ from models that do not account for the error structure. This is due to the inclusion of some pharmacies that received only 1 case combined with the fact that at low proportions, small differences in the proportions can lead to large differences in the odds-ratios. Given the consistency of the estimates across all specifications, our inability to reject the validity of the assumptions required for the random-intercepts model, and the higher precision of the model, we report results from the random intercept model shown in Column (6) in the main text.

6.1 Accounting for SP characteristics

In Appendix Table A7, we reproduce the results from Figure 1, alongside a second set of results that controls for the visible SP characteristics that could be correlated with pharmacist behavior – sex, age, height and weight. As can be seen from the table below, the 95% confidence intervals for the model with SP characteristics included indicate that the estimated odds ratios do not significantly differ from those

reported in our main specification, leading us to believe that any confounding due to SP effects is statistically insignificant as well as small in absolute magnitude. For ideal case management and referral, the point estimates of the odds-ratios are larger, but again, this reflects the low proportions and small changes as seen by the overlapping confidence intervals.

Appendix Table A7. Summary of differences between Case 1 and Case 2, controlled for SP characteristics

	(1)	(2)	(3)	(4)	(5)	(6)	(7)	(8)
	Final Model				Controlled for SP Characteristics			
**Variable**	**Odds Ratio**	**95% Lower Bound**	**95% Upper Bound**	**P-Value**	**Odds Ratio**	**95% Lower Bound**	**95% Upper Bound**	**P-Value**
**Ideal Case Management**	21.03	12.33	35.86	0.0000	38.70	17.63	84.93	0.0000
**Referral**	16.40	10.35	25.98	0.0000	23.53	12.31	44.96	0.0000
**Medication**	0.05	0.03	0.09	0.0000	0.03	0.01	0.06	0.0000
**Antibiotic**	0.21	0.15	0.31	0.0000	0.19	0.11	0.33	0.0000
**Fluoroquinolone**	0.31	0.18	0.53	0.0000	0.31	0.14	0.66	0.0024
**Schedule H**	0.15	0.11	0.21	0.0000	0.11	0.07	0.18	0.0000
**Schedule H1**	0.44	0.23	0.82	0.0099	0.37	0.14	0.95	0.0379
**Steroid**	0.27	0.14	0.53	0.0001	0.19	0.07	0.48	0.0005

7. Using the same SPs to re-examine differences across cities

In the text, we pointed to mean differences across cities in several outcome variables. A potential confounder could be that the use of different SPs in the three cities led to different results. Fortunately, we are able to directly test for this by using the subsample of interactions where the same SP was used in multiple cities. When we do so, controlling for the case and the SP's identity, we find that Delhi and Patna were statistically indistinguishable at the 5% level of significance on every outcome save for fluoroquinolone use, which could not be computed in logistic regression since none were observed in Delhi.

Compared to Mumbai, Delhi was again statistically indistinguishable. Note, however, that the estimated differences remain of substantial magnitude; however, the reduced sample size due to the rigorous matching dramatically reduces the precision of these estimates. In this case it was impossible to calculate odds-ratios for fluoroquinolone and steroid use since no fluoroquinolones were observed in Delhi and no steroids were observed in the matching subsample of Mumbai data.

Comparing Mumbai and Patna, where the sample size of overlapping SPs is substantial, we found that pharmacies in Patna used dramatically more antibiotics, fluoroquinolones, Schedule H1 medications, and steroids for the same SP presenting the same case. However, pharmacies in Patna were not significantly more or less likely to manage cases correctly, refer SPs to other providers, or use medication in general for the same SPs presenting the same cases. The full details are summarized in Appendix Table A8.

Appendix Table A8. Differences in case management between cities, controlled for SP case and identity

Variable	(1)<br>Delhi	(2)<br>Patna	(3)<br>Mumbai	(4)<br>Overall
**Ideal Case Management**	53/104 (0.51)	194/500 (0.39)	205/596 (0.34)	452/1200 (0.38)
**Referral**	60/104 (0.58)	215/500 (0.43)	222/596 (0.37)	497/1200 (0.41)
**Medication**	58/104 (0.56)	290/500 (0.58)	416/596 (0.7)	764/1200 (0.64)
**Antibiotic**	31/104 (0.3)	193/500 (0.39)	95/596 (0.16)	319/1200 (0.27)
**Fluoroquinolone**	0/104 (0)	76/500 (0.15)	8/596 (0.01)	84/1200 (0.07)
**Schedule H**	50/104 (0.48)	216/500 (0.43)	323/596 (0.54)	589/1200 (0.49)
**Schedule H1**	9/104 (0.09)	42/500 (0.08)	5/596 (0.01)	56/1200 (0.05)
**Steroid**	11/104 (0.11)	46/500 (0.09)	4/596 (0.01)	61/1200 (0.05)

	Odds Ratio	95% Lower Bound	95% Upper Bound	P-Value
**Patna : Delhi (Odds ratios greater than 1 favor Patna)**				
**Ideal Case Management**	0.7240	0.3541	1.4806	0.3763
**Referral**	0.6701	0.3382	1.3277	0.2511
**Medication**	0.7721	0.3880	1.5365	0.4613
**Antibiotic**	1.4114	0.7221	2.7586	0.3136
**Fluoroquinolone**	n/a			
**Schedule H**	0.5705	0.2943	1.1057	0.0964
**Schedule H1**	0.6503	0.2442	1.7320	0.3892
**Steroid**	0.6882	0.2686	1.7631	0.4363
**Delhi : Mumbai (Odds ratios greater than 1 favor Delhi)**				
**Ideal Case Management**	1.9545	0.7402	5.1613	0.1762
**Referral**	2.0437	0.7578	5.5114	0.1579
**Medication**	0.7753	0.3244	1.8530	0.5670
**Antibiotic**	2.1217	0.8788	5.1223	0.0944
**Fluoroquinolone**	n/a			
**Schedule H**	1.0833	0.4752	2.4695	0.8490
**Schedule H1**	4.8000	0.6027	38.2299	0.1384
**Steroid**	n/a			
**Patna : Mumbai (Odds ratios greater than 1 favor Patna)**				
**Ideal Case Management**	0.9954	0.5434	1.8232	0.9881
**Referral**	1.5124	0.8566	2.6701	0.1538
**Medication**	0.6737	0.3948	1.1497	0.1475
**Antibiotic**	4.7785	3.0754	7.4246	0.0000
**Fluoroquinolone**	33.2856	7.9153	139.9730	0.0000
**Schedule H**	0.6745	0.4468	1.0182	0.0609
**Schedule H1**	9.5375	2.8050	32.4294	0.0003
**Steroid**	18.8219	4.3794	80.8932	0.0001

Notes: This analysis uses the subsample of interactions for each city pair for which the same individuals presented SP cases in both cities. For Patna-Delhi, N = 63 Delhi interactions and 229 Patna interactions by six SPs who worked in both cities; for Delhi-Mumbai, N = 35 Delhi interactions and 141 Mumbai interactions by three SPs who worked in both cities; and for Patna-Mumbai, N = 240 Patna interactions and 227 Mumbai interactions by four SPs who worked in both cities. Regressions are controlled for case and SP identity using fixed effects.

References

1. Office of the Registrar General & Census Commissioner India. Census of India 2011. 2011. http://censusindia.gov.in/ (accessed 9/12/2015 2015).
2. Aakar Mumbai, Access for all, ADAPT, et al. People's Vision Document for Mumbai's Development Plan (2014-2034), 2013.
3. Das J, Kwan A, Daniels B, et al. Use of standardised patients to assess quality of tuberculosis care: a pilot, cross-sectional study. *The Lancet Infectious Diseases* 2015; **15**(11): 1305-13.
4. Government of India Ministry of Health and Family Welfare. The Drugs and Cosmetics Rules, 1945 (as corrected up to the 30th November, 2004). In: India CGo, editor. New Delhi; 1945.
5. Dean A, Sullivan K, Soe M. OpenEpi: Open Source Epidemiologic Statistics for Public Health, Version. www.OpenEpi.com, updated 2015/05/04. 2015. www.OpenEpi.com (accessed June 13, 2016).
